# Diseases of Children

**Published:** 1891-08

**Authors:** 

**Affiliations:** Atlanta, Ga.


### DISEASES OF CHILDREN.

BY DR. VAN GOIDTSNOVEN, OF ATLANTA, GA.

I do not propose to worry your readers—the majority of whom are practical men and experienced physicians—by lecturing them on the subject headed above. I am sure they know at least as much as I do. But you have asked me for an item, and as human "chicks" are my hobby, I will cheerfully comply.

In nine cases out of ten, "from the mouth to the rectum," should be the physician's itinerary in his diagnostical peregrinations through the infantile geography. If your readers can see, in the following formulæ anything that can be of any service to them and prove ultimately as gratifying to them as it has been to me, I shall esteem myself a justifiable intruder.

In Thrash, Opthœ, Stomatitis:

1. ℞. Acidi Borici, f ʒ i.

Glycerini, f ℥i.

M. S.: Apply frequently over the affected parts with a cotton pencil. Also, internally.

2. ℞. Lig. Lactopeptini.

Syrupi Rhei Aromatici.

Magnesœ Lactis, (Phillip's) a. a. f ℥ i.

Acidi Borici, grs. xlv iii.

M. S.: From 20 to 60 drops according to age: p. r. n.

This is one of my favorite prescriptions. I use it in almost every form of gastric and intestinal disturbance. I doubt whether as a digestive, an aperient, or antacid and an antiseptic, this compound has a superior.

IN TEETHING.

The process of dentition, although essentially a physiological act, is but too often rendered pathological, by over-feeding or mal-nutrition. The irritation and pain occasioned by the pressure of the tooth against the sensitive dental nerves produce functional derangement in almost every organ of the body; and thus the brain, stomach, lungs, liver, bowels, and in fact every organ and function may separately and combined, become reflexedly affected through this cause. Under those circumstances, Bromides are indicated. In my humble opinion, the monobromide of camphor is the remedy par excellence and I prescribe it as follows:

3. ℞. Camphoræ monobromatœ, gr. i—ij.
Extracti Hyosciami fluidi, gtts. v—viij.
Syrupi Lactucarii, (Aubergiers), f ℥ii.

M. Signa.: One (1) teaspoonful p. r. n. This is "my soothing syrup" for babies and I use no other. I never hesitate lancing the gums when it is especially indicated. It should be done "secundum artem," not with the asinine view of helping the tooth out, but simply and rationally to relieve the tension.

This prescription, together with formula No. 2, properly alternated, seldom fails to subdue the nervous irritation and to correct the diarrhœal discharges incidental to teething.

In persistent and severe forms I prescribe thus:

4 ℞. Camphoræ monobromatæ, grs. x ij.
Salol, gr. ix—xii.
Hydrarg yri cum creta, gr x ij.
Pulv. Lactopeptini, gr. xii—xviij.

M. Triturate thoroughly and divide into twelve (12) powders. S.: One (1) powder every four (4) hours, also.

5. ℞. Spiritus frumenti, gtt. 6—20.
Aquæ lactis saccharatœ, f ʒi.

M. S.: As a dose, every four (4) hours.

These last two (2) prescriptions should be alternately administered in such a manner that two (2) hours intervene between each.

[TO BE CONTINUED.]

---

TREATMENT OF BURNS.—Rottenberg (*Therapeutische Monatshefte*, March, 1891) employs the following treatment: Blisters are not opened, but are pierced with a silk thread, soaked in sublimate solution and left in place. The whole burned area is then spread with a ten per cent iodoform-vaseline, and is covered with gummed paper or siik; the salve should be renewed daily. By this plan pain is relieved at once, and cicatrical contraction is rare.—*Ex.*

